# Achados Ecocardiográficos em Pacientes com COVID-19 com e sem Doença Cardiovascular Prévia

**DOI:** 10.36660/abc.20201300

**Published:** 2021-11-22

**Authors:** Silvio Henrique Barberato, Rafael Borsoi, Fabio Roston, Hudson Laerte Machado Miranda, Pedro Patriota, Maria Estefania Otto, Adenalva Lima de Souza Beck, Anderson da Costa Armstrong, João Marcos Bemfica Barbosa Ferreira, Ana Cristina Camarozano, Letícia Braga Paciello da Silva, Marcos Valério Coimbra Resende, Marcelo Luiz Campos Vieira, Miguel Morita Fernandes-Silva

**Affiliations:** 1 Quanta Diagnóstico – Ecocardiografia Curitiba PR Brasil Quanta Diagnóstico – Ecocardiografia, Curitiba, PR – Brasil; 2 CardioEco Centro de Diagnóstico Cardiovascular Curitiba PR Brasil CardioEco Centro de Diagnóstico Cardiovascular, Curitiba, PR – Brasil; 3 Hospital de Clínicas da Universidade Federal do Paraná Curitiba PR Brasil Hospital de Clínicas da Universidade Federal do Paraná, Curitiba, PR – Brasil; 4 Hospital Universitário da Universidade Estadual de Londrina Londrina PR Brasil Hospital Universitário da Universidade Estadual de Londrina, Londrina, PR – Brasil; 5 Hospital Samel Manaus AM Brasil Hospital Samel, Manaus, AM – Brasil; 6 Hospital Universitário da Universidade Federal do Vale do São Francisco Petrolina PE Brasil Hospital Universitário da Universidade Federal do Vale do São Francisco, Petrolina, PE – Brasil; 7 Instituto de Cardiologia do Distrito Federal Brasília DF Brasil Instituto de Cardiologia do Distrito Federal (ICDF), Brasília, DF – Brasil; 8 Hospital Sirio Libanês Brasília DF Brasil Hospital Sirio Libanês, Brasília, DF – Brasil; 9 Hospital Santa Lúcia de Brasília Brasília DF Brasil Hospital Santa Lúcia de Brasília, Brasília, DF – Brasil; 10 Universidade do Estado do Amazonas Manaus AM Brasil Universidade do Estado do Amazonas, Manaus, AM – Brasil; 11 Hospital Nossa Senhora das Graças Curitiba PR Brasil Hospital Nossa Senhora das Graças, Curitiba, PR – Brasil; 12 Hospital Samaritano São Paulo SP Brasil Hospital Samaritano, São Paulo, SP – Brasil

**Keywords:** Síndrome Respiratória Aguda Grave/complicações, SARS-CoV-2/complicações, Coronavirus-19/complicações, Pandemia, Função Cardíaca, Doenças Cardiovasculares/complicações, Insuficiência Cardíaca, Ecocardiografia/métodos, Mortalidade, Comorbidade

## Introdução

A doença do coronavírus-2019 (COVID-19), causada pelo vírus da síndrome respiratória aguda grave coronavírus 2 (SARS-CoV-2), pode resultar em insuficiência respiratória grave e lesão cardíaca aguda. Disfunção cardíaca e/ou doença cardiovascular (DCV) prévia em pacientes com COVID-19 estão associadas a pior prognóstico.^1^ A ecocardiografia transtorácica (ETT) tem um papel central no manejo dos pacientes, pois fornece uma avaliação crucial das anormalidades da função e estrutura cardíacas que impactam em seu prognóstico e tratamento.^2^ Estudos relataram taxas variadas de disfunção do ventrículo esquerdo (VE) e ventrículo direito (VD), mas não está claro com que frequência a disfunção cardíaca resulta diretamente da COVID-19.^3 – 6^ Descrevemos a prevalência dos principais achados ecocardiográficos anormais em pacientes hospitalizados com COVID-19 com e sem doença cardiovascular DCV prévia através de um estudo colaborativo multicêntrico do mundo real (Registro Brasileiro de Ecocardiografia durante a pandemia de COVID-19, ou ECOVID).

## Métodos

O ECOVID é um estudo observacional multicêntrico prospectivo de pacientes hospitalizados com COVID-19 no Brasil, iniciado em 4 de abril de 2020, ao coletar dados clínicos e ecocardiográficos das cinco macrorregiões do país. A descrição completa dos métodos do estudo foi detalhada no Material Suplementar . Resumidamente, pacientes hospitalizados consecutivos (>18 anos) com COVID-19 confirmado ou altamente provável foram incluídos. Em cada centro participante, os dados clínicos foram obtidos através dos prontuários médicos e entrevista dos pacientes com cardiologistas, e as medidas ecocardiográficas foram obtidas localmente. Os resultados foram registrados através de um formulário online de relato de caso. A maioria dos exames ecocardiográficos usou um protocolo focado, a fim de diminuir o risco para o profissional de saúde.^7^ A aquisição e interpretação das imagens foram realizadas por médicos certificados, de acordo com as diretrizes internacionais.^8 , 9^ Especificamente, a disfunção sistólica do VE foi definida pela fração de ejeção do VE (FEVE) <50% (discreta entre 40-49%; moderada entre 30-39% e grave <30%). A disfunção diastólica do VE, a disfunção sistólica do VD e a pressão sistólica da artéria pulmonar (PSAP) foram definidas e classificadas de acordo com as diretrizes (consulte o Material Suplementar ). Os achados ecocardiográficos foram resumidos de acordo com a história de DCV prévia, como definida por obstrução ≥50% em qualquer artéria coronária demonstrada por angiotomografia coronariana ou angiografia coronária, revascularização coronária, infarto do miocárdio, insuficiência cardíaca ou fibrilação atrial. Este estudo foi aprovado pelo comitê de ética do centro coordenador (# 4.033.139) e pelos comitês de ética locais de cada centro participante.

### Análise estatística

As variáveis contínuas foram apresentadas como média ± desvio padrão. A distribuição Gaussiana dos dados foi analisada observando-se a forma da distribuição, assimetria, curtose e utilizando o teste de Kolmogorov-Smirnov. Os dados categóricos foram expressos como contagens e porcentagens. Os parâmetros clínicos, demográficos e ecocardiográficos foram comparados entre os indivíduos com e sem história de DCV prévia através do teste *t* de Student não pareado ou teste do qui-quadrado, conforme apropriado. Consideramos valores de p <0,05 como estatisticamente significativos. As análises estatísticas foram realizadas no software Stata versão 15.1 (Stata Corp, College Station, TX).

## Resultados

Foram incluídos 223 pacientes hospitalizados entre 4 de abril e 9 de setembro de 2020, com idade de 61,4 ± 15,3 anos (variação de 19 a 94 anos), 59% homens, 83% com COVID-19 confirmado por RT-PCR, e 17% com COVID-19 altamente provável . As principais indicações clínicas para encaminhamento para ecocardiografia foram suspeita de insuficiência cardíaca (50%), suspeita de síndrome coronariana aguda (dor torácica, anormalidades no eletrocardiograma e elevação da troponina) (20%), instabilidade hemodinâmica (18%), suspeita de miocardite (16%), suspeita de embolia pulmonar (6%), arritmias clinicamente relevantes (5%) e outros (como suspeita de derrame pericárdico, endocardite, síncope e fonte cardioembólica de acidente vascular cerebral) (5%).

A Tabela 1 resume os dados demográficos, características clínicas e comorbidades da população. Pacientes sem DCV prévia eram mais jovens e tinham menor prevalência de fatores de risco cardiovascular, como hipertensão, diabetes e tabagismo, e eram menos propensos a ter doença pulmonar obstrutiva crônica e doença renal crônica, quando comparados com pacientes com DCV prévia ( Tabela 1 ). Os sintomas e medidas de suporte relacionados ao COVID-19 foram semelhantes entre os pacientes sem e com DCV prévia ( Tabela suplementar 1 ).

**Tabela 1:** Dados demográficos e comorbidades em pacientes hospitalizados com COVID-19 de acordo com história de doença cardiovascular prévia

	Todos os pacientes	Sem DCV anterior	Com DCV anterior	p-valor
	n=223	n=173	n=50	
Idade, anos	61,4± 15,3	59 ± 15	68 ± 14	<0,001
Sexo masculino, n (%)	132 (59,2%)	103 (59,5%)	29 (58,0%)	0,85
IMC, Kg/m^2^	27,6± 5,0	27,6 ± 5,3	27,5 ± 3,6	0,83
Obesidade, n (%)	60 (26,9%)	49 (28,3%)	11 (22,0%)	0,37
Hipertensão, n (%)	115 (51,6%)	78 (45,1%)	37 (74,0%)	<0,001
Diabetes mellitus, n (%)	77 (34,5%)	47 (27,2%)	30 (60,0%)	<0,001
Tabagismo, n (%)	30 (13,5%)	17 (9,8 %)	13 (26,0%)	0,003
DAC prévia, n (%)	30 (13,5%)		30 (60,0%)	ND
IC prévia, n (%)	16 (7,2%)		16 (32,0%)	ND
FA prévia, n (%)	9 (4,0%)		9 (18,0%)	ND
Doença pulmonar, n (%)	24 (10,8%)	14 (8,1 %)	10 (20,0%)	0,017
Doença Renal Crônica, n (%)	28 (12,6%)	17 (9,8 %)	11 (22,0%)	0,022
Diálise, n (%)	3 (1,3%)	3 (1,7 %)	0 (0,0 %)	0,35
Doença Cerebrovascular, n (%)	7 (3,1%)	4 (2,3 %)	3 (6,0 %)	0,19
Câncer, n (%)	5 (2,2%)	2 (1,2 %)	3 (6,0 %)	0,042

DCV: doença cardiovascular; IMC: índice de massa corporal; DAC: doença arterial coronariana; IC: insuficiência cardíaca; FA: fibrilação atrial; DPOC: doença pulmonar obstrutiva crônica.

**Figura 1:**
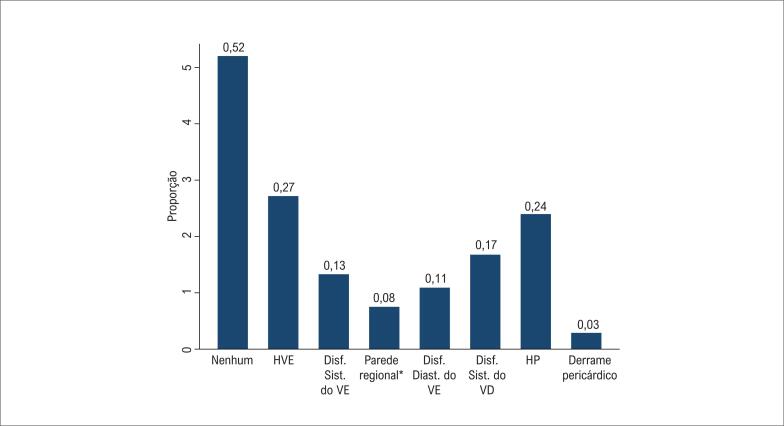
Achados ecocardiográficos em pacientes hospitalizados com COVID-19 sem doença cardiovascular prévia. HVE: hipertrofia do ventrículo esquerdo; VE: ventrículo esquerdo; VD: ventrículo direito; HP; hipertensão pulmonar. * Refere-se à anormalidade da contratilidade regional do VE. † A disfunção diastólica do VE inclui apenas disfunção diastólica do VE moderada ou grave.

A Tabela 2 mostra os principais achados ecocardiográficos em pacientes hospitalizados com COVID-19 de acordo com história de DCV prévia. Como esperado, os pacientes sem DCV eram menos propensos a apresentar achados ecocardiográficos sugerindo estrutura e / ou função do VE anormais, incluindo hipertrofia do VE (27 vs. 52%, p <0,001), disfunção sistólica do VE (13 vs. 34%, p <0,001), anormalidades da contratilidade regional (8 vs. 24%, p <0,001) e disfunção diastólica do VE grau II ou III (11 vs. 26%, p = 0,011). Por outro lado, apenas 52% dos pacientes sem DCV prévia apresentavam ecocardiograma normal ( Figura 1 ). A disfunção sistólica do VD (17 vs. 22%, p = 0,40) e hipertensão pulmonar (24 vs. 38%, p = 0,06) foram relativamente comuns e semelhantes entre pacientes sem e com DCV prévia. A disfunção sistólica do VD também foi comum em pacientes sem doença pulmonar prévia (15 vs. 20% para pacientes sem e com DCV prévia, respectivamente, p = 0,45). Derrame pericárdico e regurgitação valvar moderada a grave foram incomuns. Digno de nota, entre os pacientes sem DCV prévia e presumivelmente nova disfunção sistólica do VE (n = 21), 48% deles apresentaram anormalidades da contratilidade regional. Nenhum paciente apresentou evidência de anormalidades regionais sugestivas de cardiomiopatia induzida por estresse. Os resultados da ecocardiografia mudaram o manejo clínico em 25% dos casos, principalmente desencadeando o início da terapia para insuficiência cardíaca ou anticoagulação ou encaminhamento para cateterismo.

**Tabela 2:** Achados ecocardiográficos em pacientes hospitalizados com COVID-19 de acordo com história de doença cardiovascular prévia

Parâmetro	Todos os pacientes	Sem DCV anterior	Com DCV anterior	p-valor
	n=223	n=173	n=50	
Hipertrofia do VE, n(%)	73 (32,7%)	47 (27,2%)	26 (52,0%)	<0,001
Dilatação do VE, n(%)	31 (14,0%)	13 (7,6%)	18 (36,0%)	<0,001
Disfunção sistólica do VE, n(%)				0,005
Nenhuma	183 (82,1%)	150 (86,7%)	33 (66,0%)	
Discreta	10 (4,5%)	7 (4,0%)	3 (6,0%)	
Moderada	14 (6,3%)	8 (4,6%)	6 (12,0%)	
Grave	16 (7,2%)	8 (4,6%)	8 (16,0%)	
Disfunção diastólica do VE, n(%)				<0,001
Nenhuma	88 (42,5%)	82 (49,7%)	6 (14,3%)	
Discreta	90 (43,5%)	65 (39,4%)	25 (59,5%)	
Moderada	27 (13,0%)	17 (10,3%)	10 (23,8%)	
Grave	2 (1,0%)	1 (0,6%)	1 (2,4%)	
Desconhecida	16 (7,2%)	8 (4,6%)	8 (16,0%)	
Anormalidade da contratilidade regional do VE, n(%)	25 (11,2%)	13 (7,5%)	12 (24,0%)	0,001
Disfunção do VD, n(%)				0,20
Nenhuma	183 (82,1%)	144 (83,2%)	39 (78,0%)	
Discreta	21 (9,4%)	17 (9,8%)	4 (8,0%)	
Moderada	9 (4,0%)	7 (4,0%)	2 (4,0%)	
Grave	10 (4,5%)	5 (2,9%)	5 (10,0%)	
Hipertensão pulmonar				0,06
Nenhuma	160 (72,4%)	129 (75,4%)	31 (62,0%)	
Discreta	36 (16,3%)	28 (16,4%)	8 (16,0%)	
Moderada	21 (9,5%)	12 (7,0%)	9 (18,0%)	
Grave	4 (1,8%)	2 (1,2%)	2 (4,0%)	
Regurgitação valvar moderada ou grave, n(%)				
Aórtica	2 (0,9%)	1 (0,6%)	1 (2,0%)	0,34
Mitral	10 (4,5%)	6 (3,5%)	4 (8,2%)	0,16
Tricúspide	8 (3,6%)	4 (2,3%)	4 (8,2%)	0,05
Derrame pericárdico, n(%)	5 (2,2%)	5 (2,9%)	0 (0,0%)	0,22

DCV: doença cardiovascular; VE: ventrículo esquerdo; VD: ventrículo direito.

## Discussão

Neste registro multicêntrico, observamos que anormalidades clinicamente relevantes na função ou estrutura cardíacas foram relativamente comuns em pacientes hospitalizados com COVID-19, mesmo entre aqueles sem DCV prévia, com cerca de metade apresentando pelo menos um achado anormal. Além disso, um em cada oito pacientes sem DCV prévia apresentava pelo menos uma anormalidade ecocardiográfica grave.

Estudos anteriores descrevendo achados ecocardiográficos em pacientes com COVID-19 foram consideravelmente heterogêneos. A prevalência de disfunção sistólica do VE, disfunção do VD e dilatação do VD variou de 5,4^10^ a 37,4%,^4^ 3,6,^11^ a 33%,^12^ e 0,12 a 46,9%,^13^ respectivamente. Essa grande variação pode estar relacionada a viés de referência, diferentes protocolos de ETT, definições imprecisas de anormalidades ecocardiográficas e diferenças nas características da população, como a proporção de pacientes com DCV prévia. A fim de diminuir o viés de referência, Szekely et al.,^5^ realizaram sistematicamente o ETT em 100 pacientes consecutivos hospitalizados por COVID-19, 43% dos quais tinham DCV prévia. Eles encontraram como alteração mais frequente disfunção/dilatação do VD, enquanto apenas uma minoria dos pacientes (10%) apresentava disfunção sistólica do VE.^5^

Nosso estudo chama a atenção para a importância da DCV prévia na prevalência de achados ecocardiográficos de pacientes hospitalizados com COVID-19. Enquanto a disfunção do VD foi comum e aparentemente não relacionada à prevalência de DCV prévia, a disfunção sistólica e diastólica do VE foram mais comuns em pacientes com DCV prévia, provavelmente devido em parte a condições cardiovasculares pré-existentes. É digno de nota que 13% dos pacientes sem DCV tinham disfunção sistólica do VE, o que pode refletir um comprometimento “ *de novo* ” do VE relacionado à COVID-19. Por outro lado, a hipertensão pulmonar e a disfunção sistólica do VD têm maior probabilidade de resultar de uma miríade de fenômenos que afetam os pulmões, como hipóxia, inflamação, síndrome do desconforto respiratório agudo, trombose microvascular pulmonar, tromboembolismo pulmonar e ventilação mecânica.

Como os principais esforços da comunidade científica visam mitigar as graves consequências para a saúde causadas pela pandemia de COVID-19, torna-se um desafio equilibrar o uso da ecocardiografia para fornecer cuidados médicos de alta qualidade sem aumentar excessivamente o risco de infecção cruzada entre profissionais de saúde e pacientes. Nossos resultados ajudam a compreender, através de um registro nacional do mundo real, quais parâmetros da função cardíaca são mais frequentemente acometidos em pacientes hospitalizados com COVID-19, de acordo com a história de DCV prévia. É importante enfatizar que a presença de disfunção cardíaca é independentemente associada a um pior prognóstico em pacientes com COVID-19.^14^ A avaliação por ETT deve ser considerada em pacientes com COVID-19 e suspeita de complicações cardiovasculares para caracterizar o substrato cardíaco subjacente, para estratificação de risco, e para potencialmente orientar as estratégias de manejo.^14^ Por outro lado, suas indicações devem ser baseadas na consideração crítica dos benefícios para o paciente, risco de contaminação para profissionais de saúde e uso limitado de equipamento de proteção individual.

Nosso estudo tem limitações que merecem atenção. Primeiro, as medidas ecocardiográficas foram realizadas por investigadores locais sem avaliação final por um laboratório central. Apesar disso, todos os ecocardiogramas foram realizados por médicos experientes, que seguiram os procedimentos de acordo com as diretrizes internacionais. Em segundo lugar, os achados anormais podem ter sido superestimados devido ao viés de encaminhamento, uma vez que os ecocardiogramas foram realizados a critério do médico assistente. Terceiro, os biomarcadores séricos de lesão miocárdica não estavam disponíveis neste estudo. Finalmente, embora tenhamos descrito os achados de ETT em pacientes sem DCV prévia, ainda assim não é possível descartar se essas anormalidades cardíacas eram pré-existentes e esses resultados devem ser interpretados com cautela.

## Conclusões

Entre os pacientes hospitalizados com COVID-19 submetidos ao ecocardiograma, a disfunção sistólica do VD e do VE foi encontrada em quase um de cada cinco pacientes, mas esta última foi menos comum entre aqueles sem DCV prévia. Somente metade dos pacientes sem DCV prévia apresentou um ETT normal.

## *Material suplementar

Para informação adicional, por favor, clique aqui:


