# Supplementary material for: Achados Ecocardiográficos em Pacientes com COVID-19 com e sem Doença Cardiovascular Prévia
Source: Arq Bras Cardiol. 2021 Nov 22;117(6):1207–11. [Article in Portuguese] doi: 10.36660/abc.20201300 (PMC8757145; doi:10.36660/abc.20201300)
Supplement: Supplementary file 1 [file 2020-1300_CC_portugues_supplemental_material.pdf]

Supplemental Material

Methods

The ECHOVID registry included consecutive patients older than 18 years of age with confirmed or highly probable COVID-19 who were referred to TTE during hospital stay. Patients with clinical symptoms of COVID-19 and real-time polymerase chain reaction (RT-PCR) positive tests were classified as confirmed cases. Patients with clinical signs and symptoms of COVID-19 and compatible findings on chest computed tomography (bilateral ground-glass opacities), but without a RT-PCR test, were considered as highly probable cases. This study was approved by the ethics committee of the coordinating center (# 4.033.139) and the local ethics committees from each respective site. To reduce the risk of SARS-CoV-2 cross-infection between healthcare workers and patients, the ethics committee waived the requirement to obtain a signed consent form and approved entry of unidentified patient information into the study database.

Data collection

At each participant center, the cardiologists completed a case report form after the echocardiogram. The protocol definitions in the case report form were discussed with each investigator, who were asked to follow the same standard to improve consistency across sites. Data were entered online (https://forms.gle/g4NXJir6tHR8dGMZ7) and submitted to the coordinating center via a secure website. The study coordinating center assigned a unique identification number for each patient to avoid duplicate entries and certify the security of protected health information.

Clinical and Demographic data

Each investigator reviewed medical charts and interviewed the patient or relative to obtain clinical and demographic data, including age, sex, weight, height, history of arterial hypertension, diabetes mellitus, obesity, smoking, coronary artery disease (prior myocardial infarction, percutaneous coronary intervention and/or coronary artery bypass graft) heart failure, atrial fibrillation, chronic obstructive pulmonary disease, cerebrovascular stroke, chronic kidney disease (serum creatinine level ≥ 1.5 mg/dL) and cancer. Previous CVD was defined as history of any of the following: coronary computed tomography angiography or coronary angiography showing an obstruction >=50% in any major coronary artery, percutaneous or surgical coronary revascularization, myocardial infarction, heart failure or atrial fibrillation. COVID-19-related signs and symptoms and the need for supportive measures in critically ill patients (non-invasive ventilation, mechanical ventilation and use of vasoactive drugs) were also collected.

Echocardiographic data

In order to mitigate the risk to the healthcare professional, a focused protocol was used in the majority of the scans.[1, 2] Imaging acquisition and interpretation were performed by certified physicians according to international guidelines.[3, 4] All procedures followed the respective institutional protocol regarding personal protective equipment (PPE) and equipment cleaning after each scan. LV ejection fraction (LVEF) was assessed by visual estimation and/or modified Simpson's biplane. LV systolic dysfunction was defined by LVEF below 50% (mild between 40-49%; moderate between 30-39%, and severe < 30%). LV diastolic function

analysis included measurements of peak early filling (E-wave) and late diastolic filling (A-wave) velocities, E/A ratio, early diastolic mitral septal and lateral annular velocities (e´), and E/e´ ratio. Patients were classified as having normal (E/A 0.8–2.0, E/e´< 10) or abnormal diastolic dysfunction (grade I dysfunction: E/A ≤ 0.8, E/e´< 10; grade II dysfunction: E/A 0.8–2.0, E/e´10-14; or grade III dysfunction: E/A ≥ 2; E/e´> 14).[(5)] Diastolic function grade was considered "unknown" when it was judged to be "indeterminate" or when data were not collected. RV systolic dysfunction was determined by visual estimation and/or semi-quantitatively (tricuspid annular plane systolic excursion [TAPSE] < 17mm and/or tissue Doppler of the free lateral wall [S'] < 9.5cm/s indicate RV dysfunction).[6] Valvular regurgitation was assessed as per by the guidelines.[7] Pulmonary artery systolic pressure (PASP) was estimated from the peak tricuspid regurgitation velocity, obtained by continuous-wave Doppler echocardiography, and the right atrial pressure estimated by inferior vena cava size. Pulmonary hypertension was defined as PASP ≥ 36mmHg (mild between 36-45mmHg; severe if ≥ 60mmHg).

Statistical Analysis

Continuous variables were assessed for the Gaussian distribution of the data and presented as mean ± standard deviation or median (25th percentile, 75th percentile) accordingly. Clinical, demographic and echocardiographic parameters were compared between the two groups based on the history of CVD using t-Student test or Chi-squared test. For some patients, data was missing on days since symptoms onset, fever, cough, dyspnea, myalgia, sore throat, diarrhea, headache, anosmia and chest pain. We used multiple imputation to

account for missing data, under a missing at random assumption. Data was imputed using data augmentation algorithm, a Markov Chain Monte Carlo procedure, assuming a joint multivariate normal distribution for the variables in the imputation model. [8] Imputation of 20 datasets was performed using age, sex, BMI and site center as predictors. Linear regression was then applied to the multiple-imputed data, combining the 20 datasets as previously described, in order to compare the results between patients with and without previous cardiovascular diseases.[9] We considered statistically significant p-values<0.05. Statistical analyses were performed using Stata version 15.1 (Stata Corp, College Station, TX).

Supplemental table 1. COVID-19-related symptoms and supportive measures according to history of previous cardiovascular diseases*

	All patients n=223	No previous CVD n=173	Previous CVD n=50	*p* value
Days since symptoms onset**	10.5 ± 0.5	10.9 ± 0.6	9.2 ± 1.3	0.38
Fever, n(%)	138 (61.8%)	110 (63.5%)	27 (55.3%)	0.25
Cough, n(%)	131 (58.7%)	106 (61.0%)	25 (50.8%)	0.15
Dyspnea, n(%)	163 (73.3%)	1290 (74.8%)	34 (68.1%)	0.38
Myalgia, n(%)	52 (23.3%)	43 (24.9%)	9 (17.9%)	0.27
Sore throat, n(%)	16 (7.1%)	10 (6.1%)	5 (10.5%)	0.50
Diarrhea, n(%)	26 (11.5%)	22 (12.6%)	4 (7.9%)	0.43
Headache, n(%)	24 (10.7%)	18 (10.6%)	6 (11.1%)	0.97
Anosmia, n(%)	5 (2.3%)	5 (2.9%)	0 (0.0%)	0.58
Chest pain, n(%)	13 (5.6%)	9 (5.5%)	3 (6.1%)	0.88
Intensive care, n(%)	137 (61.4%)	107 (61.8%)	30 (60.0%)	0.81
NIV, n(%)	46 (20.6%)	31 (17.9%)	15 (30.0%)	0.06
Intubated, n(%)	89 (39.9%)	74 (42.8%)	15 (30.0%)	0.10
Vasopressor drug, n(%)	28 (12.6%)	24 (13.9%)	4 (8.0%)	0.27

CVD – cardiovascular disease, NIV – Non-invasive mechanical ventilation
*We used multiple imputation for 32 patients with missing data for days since symptoms onset, fever ,cough, dyspnea, myalgia, sore throat, diarrhea, headache, anosmia and chest pain (please see Methods for details).
** Described as mean ± standard error.

References

1. Kirkpatrick JN, Mitchell C, Taub C, Kort S, Hung J, Swaminathan M. ASE Statement on Protection of Patients and Echocardiography Service Providers During the 2019 Novel Coronavirus Outbreak: Endorsed by the American College of Cardiology. J Am Soc Echocardiogr. 2020;33(6):648-53.
2. Skulstad H, Cosyns B, Popescu BA, Galderisi M, Salvo GD, Donal E, et al. COVID-19 pandemic and cardiac imaging: EACVI recommendations on precautions, indications, prioritization, and protection for patients and healthcare personnel. Eur Heart J Cardiovasc Imaging. 2020.
3. Lang RM, Badano LP, Mor-Avi V, Afilalo J, Armstrong A, Ernande L, et al. Recommendations for cardiac chamber quantification by echocardiography in adults: an update from the American Society of Echocardiography and the European Association of Cardiovascular Imaging. J Am Soc Echocardiogr. 2015;28(1):1-39.e14.
4. Douglas PS, Carabello BA, Lang RM, Lopez L, Pellikka PA, Picard MH, et al. 2019 ACC/AHA/ASE Key Data Elements and Definitions for Transthoracic Echocardiography: A Report of the American College of Cardiology/American Heart Association Task Force on Clinical Data Standards (Writing Committee to Develop Cardiovascular Endpoints Data Standards) and the American Society of Echocardiography. J Am Coll Cardiol. 2019;74(3):403-69.
5. Nagueh SF, Appleton CP, Gillebert TC, Marino PN, Oh JK, Smiseth OA, et al. Recommendations for the evaluation of left ventricular diastolic function by echocardiography. Eur J Echocardiogr. 2009;10(2):165-93.
6. Rudski LG, Lai WW, Afilalo J, Hua L, Handschumacher MD, Chandrasekaran K, et al. Guidelines for the echocardiographic assessment of the right heart in adults: a report from the American Society of Echocardiography endorsed by the European Association of Echocardiography, a registered branch of the European Society of Cardiology, and the Canadian Society of Echocardiography. J Am Soc Echocardiogr. 2010;23(7):685-713; quiz 86-8.
7. Nishimura RA, Otto CM, Bonow RO, Carabello BA, Erwin JP, Fleisher LA, et al. 2017 AHA/ACC Focused Update of the 2014 AHA/ACC Guideline for the Management of Patients With Valvular Heart Disease: A Report of the American College of Cardiology/American Heart Association Task Force on Clinical Practice Guidelines. J Am Coll Cardiol. 2017;70(2):252-89.
8. Demirtas H, Freels SA, Yucel RM. Plausibility of multivariate normality assumption when multiply imputing non-Gaussian continuous outcomes: a simulation assessment. Journal of Statistical Computation and Simulation. 2008;78(1):69-84.
9. Toutenburg H. Rubin, D.B.: Multiple imputation for nonresponse in surveys. Statistical Papers. 1990;31(1):180-.
