# Supplementary material for: Achados Ecocardiográficos em Pacientes com COVID-19 com e sem Doença Cardiovascular Prévia
Source: Arq Bras Cardiol. 2021 Nov 22;117(6):1207–11. [Article in Portuguese] doi: 10.36660/abc.20201300 (PMC8757145; doi:10.36660/abc.20201300)
Supplement: Translation

# Translation: Echocardiographic Findings in Patients with COVID-19 with and without Previous Cardiovascular Disease

**Authors:** Silvio Henrique Barberato, Rafael Borsoi, Fabio Roston, Hudson Laerte Machado Miranda, Pedro Patriota, Maria Estefania Otto, Adenalva Lima de Souza Beck, Anderson da Costa Armstrong, João Marcos Bemfica Barbosa Ferreira, Ana Cristina Camarozano, Letícia Braga Paciello da Silva, Marcos Valério Coimbra Resende, Marcelo Luiz Campos Vieira, Miguel Morita Fernandes-Silva

**Affiliations:** 1 Curitiba PR Brazil Quanta Diagnóstico – Ecocardiografia, Curitiba, PR – Brazil; 2 Curitiba PR Brazil CardioEco Centro de Diagnóstico Cardiovascular, Curitiba, PR – Brazil; 3 Curitiba PR Brazil Hospital de Clínicas da Universidade Federal do Paraná, Curitiba, PR – Brazil; 4 Londrina PR Brazil Hospital Universitário da Universidade Estadual de Londrina, Londrina, PR – Brazil; 5 Manaus AM Brazil Hospital Samel, Manaus, AM – Brazil; 6 Petrolina PE Brazil Hospital Universitário da Universidade Federal do Vale do São Francisco, Petrolina, PE – Brazil; 7 Brasília DF Brazil Instituto de Cardiologia do Distrito Federal (ICDF), Brasília, DF – Brazil; 8 Brasília DF Brazil Hospital Sirio Libanês, Brasília, DF – Brazil; 9 Brasília DF Brazil Hospital Santa Lúcia de Brasília, Brasília, DF – Brazil; 10 Manaus AM Brazil Universidade do Estado do Amazonas, Manaus, AM – Brazil; 11 Curitiba PR Brazil Hospital Nossa Senhora das Graças, Curitiba, PR – Brazil; 12 São Paulo SP Brazil Hospital Samaritano, São Paulo, SP – Brazil

**Keywords:** Acute Respiratory Syndrome/complications SARS-CoV2/complications, Coronavirus-19/complications, Pandemics, Cardiac Function, Cardiovascular Diseases/complications, Heart Failure, Echocardiography/méthods, Mortality, Comorbidity

## Introduction

Coronavirus disease-2019 (COVID-19) caused by the severe acute respiratory syndrome coronavirus 2 (SARS-Cov-2) may result in severe respiratory distress and acute cardiac injury. Impaired cardiac function and/or prior cardiovascular disease (CVD) in patients with COVID-19 are associated with worse prognosis.^1^Transthoracic echocardiography (TTE) has a central role in the management of patients, as it provides a crucial assessment of abnormalities in cardiac function and structure that impact on their prognosis and treatment.^2^Studies have reported varied rates of left ventricular (LV) and right ventricular (RV) dysfunction, but it is unclear how often cardiac dysfunction result directly from COVID-19.^3 – 6^We described the prevalence of the main abnormal echocardiographic findings in hospitalized patients with COVID-19 with and without previous cardiovascular disease (CVD) through a real-world, multicenter collaborative study (Brazilian Echocardiography Registry during COVID-19 pandemic, or ECOVID).

## Methods

ECOVID is a prospective multicenter observational study of hospitalized patients with COVID-19 in Brazil that started on April 4^th^, 2020, by collecting clinical and echocardiographic data in all five macro-regions of the country. Full description of the study methods was detailed in the Supplemental Material . Briefly, consecutive hospitalized patients (> 18 years old) with confirmed or highly probable COVID-19 were included. At each participant center, clinical data was obtained from medical charts and patient interview by cardiologists, and echocardiographic measures were locally obtained. The results were registered using an online case report form. Most echocardiographic scans used a focused protocol aiming to mitigate the risk to the healthcare professional.^7^Imaging acquisition and interpretation were performed by certified physicians according to international guidelines.^8 , 9^Specifically, LV systolic dysfunction was defined by LV ejection fraction (LVEF) below 50% (mild between 40-49%; moderate between 30-39%, and severe <30%). LV diastolic dysfunction, RV systolic dysfunction and pulmonary artery systolic pressure (PASP) were defined and classified according to guidelines (please see Supplemental material ). The echocardiographic findings were summarized according to the history of previous CVD, as defined by previous obstruction ≥50% in any major coronary artery demonstrated by coronary computed tomography angiography or coronary angiography, coronary revascularization, myocardial infarction, heart failure or atrial fibrillation. This study was approved by the ethics committee of the coordinating center (# 4.033.139) and the local ethics committees from each respective site.

### Statistical analysis

Continuous variables were presented as mean ± standard deviation. The Gaussian distribution of the data was analyzed by looking at the shape of the distribution, skewness, kurtosis, and using the Kolmogorov-Smirnov test. Categorical data were expressed as counts and percentages. Clinical, demographic and echocardiographic parameters were compared between the individuals with and without history of previous CVD using unpaired Student's *t* test or Chi-squared test, accordingly. We considered statistically significant p-values<0.05. Statistical analyses were performed using Stata version 15.1 (Stata Corp, College Station, TX).

## Results

We included 223 hospitalized patients admitted between April 4^th^and September 9^th^, 2020, aged 61.4 ± 15.3 years old (range19 to 94), 59% men, 83% with RT-PCR-confirmed COVID-19, 17% with highly probable COVID-19. The main clinical indications for referral for echocardiography were suspected heart failure (50%), suspected acute coronary syndrome (chest pain, electrocardiogram abnormalities and troponin elevation) (20%), hemodynamic instability (18%), suspected myocarditis (16%), suspected pulmonary embolism (6%), clinically relevant arrhythmias (5%), and others (such as suspected pericardial effusion, endocarditis, syncope, and cardioembolic source of brain stroke) (5%).

Table 1 summarizes the demographics, clinical characteristics and comorbidities of the population. Patients without previous CVD were younger and had lower prevalence of cardiovascular risk factors, such as hypertension, diabetes and smoking, and were less likely to have chronic obstructive pulmonary disease and chronic kidney disease, when compared with patients with previous CVD ( Table 1 ). COVID-19-related symptoms and supportive measures were similar between patients without and with previous CVD ( Supplemental table 1 ).

**Table 1:** Demographics and comorbidities in hospitalized patients with COVID-19 according to a history of previous cardiovascular disease

	All patients	No previous CVD	Previous CVD	p value
	n=223	n=173	n=50	
Age, years	61.4± 15.3	59 ± 15	68 ± 14	<0.001
Male, n (%)	132 (59.2%)	103 (59.5%)	29 (58.0%)	0.85
BMI, Kg/m^2^	27.6± 5.0	27.6 ± 5.3	27.5 ± 3.6	0.83
Obesity, n (%)	60 (26.9%)	49 (28.3%)	11 (22.0%)	0.37
Hypertension, n (%)	115 (51.6%)	78 (45.1%)	37 (74.0%)	<0.001
Diabetes mellitus, n (%)	77 (34.5%)	47 (27.2%)	30 (60.0%)	<0.001
Smoking, n (%)	30 (13.5%)	17 (9.8 %)	13 (26.0%)	0.003
Previous CAD, n (%)	30 (13.5%)		30 (60.0%)	ND
Previous HF, n (%)	16 (7.2%)		16 (32.0%)	ND
Previous AF, n (%)	9 (4.0%)		9 (18.0%)	ND
Pulmonary disease, n (%)	24 (10.8%)	14 (8.1 %)	10 (20.0%)	0.017
Chronic Kidney disease, n (%)	28 (12.6%)	17 (9.8 %)	11 (22.0%)	0.022
Dialysis, n (%)	3 (1.3%)	3 (1.7 %)	0 (0.0 %)	0.35
Cerebrovascular disease, n (%)	7 (3.1%)	4 (2.3 %)	3 (6.0 %)	0.19
Cancer, n (%)	5 (2.2%)	2 (1.2 %)	3 (6.0 %)	0.042

CVD: cardiovascular disease; BMI: body mass index; CAD: coronary artery disease; HF: Heart failure; AF: atrial fibrillation; COPD: Chronic obstructive pulmonary disease.

Table 2 shows the main echocardiographic findings in hospitalized patients with COVID-19 according to a history of previous CVD. As expected, patients without CVD were less likely to have echocardiographic findings suggesting abnormal LV structure and/or function, including LV hypertrophy (27 vs 52%, p<0.001), LV systolic dysfunction (13 vs. 34%, p<0.001), regional wall motion abnormalities (8 vs. 24%, p<0.001) and grade II or III LV diastolic dysfunction (11 vs. 26%, p=0.011). On the other hand, only 52% of patients without previous CVD had a normal echocardiogram ( Figure 1 ). RV systolic dysfunction (17 vs. 22%, p=0.40) and pulmonary hypertension (24 vs. 38%, p=0.06) were relatively common and they were similar between patients without and with previous CVD. RV systolic dysfunction was also common in patients without previous pulmonary disease (15 vs. 20% for patients without and with previous CVD, respectively, p=0.45). Pericardial effusion and moderate-to-severe valve regurgitation were uncommon. Of note, in patients without previous CVD and presumably new LV systolic dysfunction (n=21), 48% of them displayed regional wall motion abnormalities. No patient had evidence of wall motion abnormalities suggestive of stress-induced cardiomyopathy. Echocardiography results changed clinical management in 25% of the cases, mostly triggering the initiation of therapy for heart failure or anticoagulation or referral to catheterization.

**Figure 1:** Echocardiographic findings in patients hospitalized for COVID-19 without previous cardiovascular disease. LVH: left ventricular hypertrophy; LV: left ventricle; RV: right ventricle; PH: pulmonary hypertension. *Refers to LV regional wall motion abnormality. †LV diastolic dysfunction includes only moderate or severe LV diastolic dysfunction.

**Table 2:** Echocardiographic findings in hospitalized patients with COVID-19 according to a history of previous cardiovascular disease

Parameter	All patients	No previous CVD	Previous CVD	p value
	n=223	n=173	n=50	
LV hypertrophy, n(%)	73 (32.7%)	47 (27.2%)	26 (52.0%)	<0.001
LV dilation, n(%)	31 (14.0%)	13 (7.6%)	18 (36.0%)	<0.001
LV systolic dysfunction, n(%)				0.005
None	183 (82.1%)	150 (86.7%)	33 (66.0%)	
Mild	10 (4.5%)	7 (4.0%)	3 (6.0%)	
Moderate	14 (6.3%)	8 (4.6%)	6 (12.0%)	
Severe	16 (7.2%)	8 (4.6%)	8 (16.0%)	
LV diastolic dysfunction, n(%)				<0.001
None	88 (42.5%)	82 (49.7%)	6 (14.3%)	
Mild	90 (43.5%)	65 (39.4%)	25 (59.5%)	
Moderate	27 (13.0%)	17 (10.3%)	10 (23.8%)	
Severe	2 (1.0%)	1 (0.6%)	1 (2.4%)	
Unknown	16 (7.2%)	8 (4.6%)	8 (16.0%)	
LV regional wall abnormality, n(%)	25 (11.2%)	13 (7.5%)	12 (24.0%)	0.001
RV dysfunction, n(%)				0.20
None	183 (82.1%)	144 (83.2%)	39 (78.0%)	
Mild	21 (9.4%)	17 (9.8%)	4 (8.0%)	
Moderate	9 (4.0%)	7 (4.0%)	2 (4.0%)	
Severe	10 (4.5%)	5 (2.9%)	5 (10.0%)	
Pulmonary hypertension				0.06
None	160 (72.4%)	129 (75.4%)	31 (62.0%)	
Mild	36 (16.3%)	28 (16.4%)	8 (16.0%)	
Moderate	21 (9.5%)	12 (7.0%)	9 (18.0%)	
Severe	4 (1.8%)	2 (1.2%)	2 (4.0%)	
Moderate-to-severe valve regurgitation, n(%)				
Aortic	2 (0.9%)	1 (0.6%)	1 (2.0%)	0.34
Mitral	10 (4.5%)	6 (3.5%)	4 (8.2%)	0.16
Tricuspid	8 (3.6%)	4 (2.3%)	4 (8.2%)	0.05
Pericardial effusion, n(%)	5 (2.2%)	5 (2.9%)	0 (0.0%)	0.22

CVD: cardiovascular disease; LV: left ventricular; RV: right ventricular.

## Discussion

In this multicenter registry, we found that clinically relevant abnormalities in cardiac function or structure were relatively common among hospitalized patients with COVID-19, even in those without previous CVD, with roughly half showing at least one abnormal finding. Moreover, 1 in 8 patients without previous CVD had at least one severe echocardiographic abnormality.

Previous studies describing echocardiographic findings in patients with COVID-19 have been considerable heterogeneous. The prevalence of LV systolic dysfunction, RV dysfunction and RV dilation have ranged from 5.4^10^to 37.4%,^4^3.6,^11^to 33%,^12^and 0,12 to 46.9%,^13^respectively. This wide variation may be related to referral bias, different TTE protocols, inaccurate definitions of echocardiographic abnormalities, and differences in population characteristics, such as the proportion of patients with previous CVD. Aiming to mitigate referral bias, Szekely et al.^5^systematically performed TTE in 100 consecutive patients hospitalized for COVID-19, 43% of which had prior CVD. They found that the most frequent abnormality was RV dysfunction/dilation while only a minority of patients (10%) had LV systolic dysfunction.^5^

Our study sheds light on the importance of previous CVD on the prevalence of echocardiographic findings of patients hospitalized with COVID-19. While RV dysfunction was common and apparently unrelated to the prevalence of previous CVD, LV systolic and diastolic dysfunction were more common in patients with previous CVD, likely in part due to pre-existing cardiovascular conditions. Noteworthy, thirteen percent of patients without CVD had LV systolic dysfunction, which may reflect a COVID-19-related *“de novo”* LV impairment. On the other hand, pulmonary hypertension and RV systolic dysfunction are more likely to result from a myriad of phenomena that affects the lungs, such as hypoxia, inflammation, acute respiratory distress syndrome, pulmonary microvascular thrombosis, pulmonary thromboembolism and mechanical ventilation.

As major efforts by the scientific community aim to mitigate the severe health consequences of the COVID-19 pandemic, it becomes challenging to balance the use of echocardiography to provide high quality of medical care without excessively increasing the risk of cross-infection between healthcare professionals and patients. Our results help understand which cardiac function parameters are most frequently abnormal in hospitalized patients with COVID-19, according to the history of previous CVD through a real-world national registry. It is important to emphasize that the presence of cardiac dysfunction is independently associated with worse prognosis in patients with severe COVID-19.^14^TTE evaluation should be considered in patients with COVID-19 and suspected cardiovascular complications to characterize the underlying cardiac substrate, for risk stratification, and to potentially guide management strategies.^14^On the other hand, its indications should be based in critical consideration of the benefits to patient, contamination risk for healthcare personnel and use of the limited personal protective equipment.

Our study has limitations that deserve attention. First, the echocardiographic measures were performed by local investigators without final assessment by a core lab. Nevertheless, all echocardiograms were performed by experienced physicians, who followed the procedures according to international guidelines. Second, abnormal findings may have been overestimated due to referral bias, as the echocardiograms were performed at the discretion of the attending physician. Third, serum biomarkers of myocardial injury were unavailable in this study. Finally, although we described the TTE findings in patients without previous CVD, we still cannot rule out whether these cardiac abnormalities were pre-existing, and these results should be interpreted with caution.

## Conclusions

Among hospitalized patients with COVID-19 submitted to an echocardiogram, RV and LV systolic dysfunction were found in almost one out of five patients, but the latter was less common in those without previous CVD. Only half of the patients without previous CVD had a normal TTE.

## *Supplemental Materials

For additional information, please,click here:
